# Secularism as a Project of Free and Equal Citizenship: Reflections on the Turkish Case

**DOI:** 10.3389/fsoc.2022.902734

**Published:** 2022-06-15

**Authors:** Haldun Gülalp

**Affiliations:** Turkish Economic and Social Studies Foundation (TESEV), Istanbul, Turkey

**Keywords:** secularism, secularization, freedom of belief, citizenship, democracy, authoritarianism, Turkey

## Abstract

This article undertakes a defense of secularism, much maligned by postmodernists and multiculturalists. First, secularism as a normative political principle is conceptually distinguished from the discredited sociological theory of secularization and, second, it is treated as a project of free and equal citizenship. The conceptual discussion is complemented by an assessment of the Turkish case, falsely presented in the literature as a radical form of secularism. The article aims to show that a religious political movement, opposed to secularism, tends to be authoritarian and intolerant of diversity.

## Introduction

In conventional political sociology, *secularization* is conceptualized as the decline of the “social significance of religion” and theorized as an outcome of modernization (Wallis and Bruce, 1992). The reemergence of religious identities in social and political life toward the end of the twentieth century has therefore led to a questioning of this theory. The issue of *secularism*, however, should be treated separately from this theory and its predictions. As a normative political principle that aims to protect the citizens' freedom of belief, secularism is a different matter from the increase or decrease of religiosity in society, although it is of course a product of the Enlightenment and of modernity, having grown out of efforts to end the religious wars of early modern Europe. As such, it is a cornerstone of free and equal citizenship and hence the road to democracy.

Refusing secularism on the grounds that it opposes religiosity, or restricts the freedom of the religious, overlooks the indifference of normative secularism to the question of private religiosity. Secularism is not concerned with praising or rebuking religion(s), but with the protection of freedom of thought, belief and conscience of all citizens, whether they are religious or not. The critics also fail to see that opening up political space for the hierarchical and absolutist structures of religious organizations, in the name of religious freedom, is incompatible with a regime of democratic and egalitarian citizenship.

The point of secularism's protection of the freedom of conscience and belief is not to allow religion(s) to occupy the political space, but to enable citizens to have the right to believe or not as they wish, and to fulfill the requirements of their beliefs individually or collectively, provided they are not infringing on the rights of others. In short, secularism is a right of citizenship, a means to freedom. Objecting to this and making space for religion and religious organizations in the political arena would not broaden freedoms, it would narrow and eventually destroy them. Some authoritarian states may claim to defend secularism, but they are not authoritarian *because* they are secularist. If their authoritarianism involves the restriction of the freedom of religion of their citizens, they cannot be considered secular. By contrast, a state that is *not* secular is by definition authoritarian, because its government relies directly or indirectly on religious rules (as interpreted by the government), with priorities that are deemed indisputable, rather than on rules negotiated by reason.

## Secularization

The *secularization* thesis was an integral part of modernization theory that originated in the nineteenth century and dominated the social sciences for most of the twentieth. Its prediction that societies would inevitably progress toward modernity, and in conjunction would also secularize, fell apart in the late twentieth century, giving rise to postmodernism. Aside from the fallacy of the idea that modernization was inevitable and would proceed in the same way everywhere, the thesis that it would cause a decline in religiosity was problematic from the very beginning. For instance, religiosity as measured by church-going has remained high in the United States (US), although it has decreased significantly in most European countries. In response to this anomaly, various interpretations have been offered to explain US “exceptionalism.” A plausible argument has been that people's habits of going to church could not be explained by religiosity alone. Going to church was a traditional form of socialization, a means of building communal bonds and solidarity in a society dominated by individualism. Therefore, the US could be seen as an exception, leaving the main rule intact.

Nevertheless, a series of events in the late 1970's and early 1980's caused the unavoidable collapse of the secularization thesis. In the US, where individual religiosity was already prevalent, social groups based on religious identities began to *politically* mobilize and achieve a certain degree of power. In 1980, Ronald Reagan was elected president with the support of these groups. The US also chose to work with Islamist groups in Afghanistan in its struggle against the Soviet regime, contributing to religion-based political mobilization outside of its borders. A much more important event was the establishment of the Islamic Republic in Iran. The broad coalition which successfully overthrew the pro-US Shah in 1979 included leftists and liberals along with Islamists. The Islamist wing subsequently liquidated the other groups and declared its hegemony. This outcome overturned all familiar modernization and secularization theses. The establishment of an Islamist regime was a shocking outcome for those pro-modernist groups that wanted the Shah to go, but it was welcomed by such postmodernists as Michel Foucault (Afary and Anderson, 2005).

Developments in Western Europe further undermined the secularization thesis. One of the effects of neoliberal globalization was the large scale migration from Muslim countries to Europe in the post-1980 period. The growth of the Muslim population not only reversed the tendency of European societies to secularize, it also put the previously settled issue of *secularism* back on the agenda, this time in an inextricable way. Reaching a significant size, these immigrant communities that used to be known by their national origins began to be recognized by their religious identities, partly as a result of their own demands and partly as a reaction of the host societies to the large number of culturally unassimilated newcomers. As the immigrant Muslim populations became an important presence in European nations, voices began to emerge that rejected the dominant model of “separation of state and religion” and to put forward alternative demands, which were also supported by local postmodernists. Eager to defend the “right of identity groups to be recognized” they too began to question the principle of secularism in the name of “multiculturalism.” The fading of the *secularization* thesis therefore went hand in hand with the questioning of the principle of *secularism*, although it had been the classic formula for domestic peace after the long religious wars.

## Secularism

The important difference between the theory of *secularization* and the normative principle of *secularism* is best seen in the seemingly paradoxical notion that secularism aims to protect individual religious freedom. A secular state is not one that suppresses religion, but one that defines and structures itself *independently* of it, thereby granting freedom of belief to citizens. Freedom of belief naturally includes the freedom not to believe. A secular state is liberal, it does not interfere with, nurture or hinder the belief held by its citizens individually or collectively. Secularism is not an ideology; it is a project of freedom and equality for citizens, and aims to ensure it by rendering the state neutral in this realm. For the same reason, secularism does not grant religious organizations the right to have a place in the political arena, because a pathway to their sovereignty would threaten the freedom of belief that secularism aims to protect.

According to a fundamental human right, defined by both the *Universal Declaration of Human Rights* (Article 18) and the *European Convention on Human Rights* (Article 9) in the same way, “Everyone has the right to freedom of thought, conscience and religion; this right includes freedom to change his [*sic*] religion or belief and freedom, either alone or in community with others and in public or private, to manifest his [*sic*] religion or belief, in worship, teaching, practice and observance.” As noted above, the freedom to have a belief and to manifest it necessarily includes the freedom to not believe and to not be forced to publicly manifest it. This was clearly stated in the first-ever finding of a violation of Article 9 by the European Court of Human Rights (ECtHR). In the case of *Kokkinakis v. Greece*, the ECtHR noted that Article 9 of the Convention is not only vital for the religious, “it is also a precious asset for atheists, agnostics, skeptics and the unconcerned” (ECtHR, 14307/88, 25 May 1993, paragraph 31). In other decisions, the ECtHR has also ruled that States cannot compel anyone to disclose their belief (e.g., ECtHR, *Işik v. Turkey*, 21924/05, 2 February 2010).

Secularism, then, is a liberal principle that aims to protect the freedom of persons to believe in any religion or to not believe at all. It therefore implies the existence of a segregated area free of state interference where individuals and groups can enjoy their freedom of religion and conscience, provided they do not disturb the public order or violate anyone else's rights. The secular state is responsible for creating this political space, separate from and independent of religions, where all persons can negotiate their common problems as a civic community. Whether religiosity decreases or increases in society (as per *or* contra the secularization theory) the state can still consistently follow the principle of secularism.

One may question at this point whether “freedom of religion and conscience” should not include the presence of religion(s) in the political arena, as suggested by some religious circles and their postmodernist and multiculturalist supporters. What if the notion of protecting freedom of belief from state intervention leads in the opposite direction, that is, to the intervention of belief and belief communities in state affairs? In that case, would the state not have to restrain belief in the name of secularism?

There is a coherent answer to this question within the framework of normative secularism. You may certainly have political views and preferences originating from your belief and you may bring them into public debate. No one can object to your expression of ideas deriving from your belief. But this statement also implies an important limitation. Bringing these ideas into public discussion and debate cannot involve an attempt to go beyond expressing them, such as trying to impose the prescriptions or requirements of your belief on others. While as a citizen with the same rights as you I am obligated to respect your adherence to a certain belief, in no way do I have the responsibility to respect the contents of your belief (even if it is shared by the majority) or to regard it as sacred as you do, because I may have a different belief of my own, or no belief at all, which is (or ought to be) equally protected. Your faith may be sacred to you, but to someone else their own faith is. No one can humiliate or harm you because of your belief, but no one has to share your belief or be expected to conform to its rules. The belief you hold individually (or collectively with others) may guide you in your own world, but it does not concern anyone else. In public negotiation with others, you need to persuade them with logic and reasoning, and not by threats, intimidation, or the mention of sacred red lines that cannot be crossed.

## State and Religion

What structural form should secularism take in the organization of the state? In reference to the original meaning of the term secularism itself, that is, the realm outside of religion (or the sphere of non-clerical matters), the simplest conception of this form is the separation of political authority and religious authority. Those who believe in a particular religion may, if they wish, follow the rules and guidance of the clerical authority of that religion; but they cannot oblige anyone else to do the same, those rules cannot go beyond helping them regulate their own private lives. The political sphere where citizenship resides is a different and independent matter. Likewise, political authority cannot interfere with the belief or disbelief of citizens, or the respect that they may or may not have for religious authorities, as long as they do not violate anyone else's human rights.

The conceptual distinction between secularization and secularism is also useful here. The *secularization* of state administration is not the same as the state's upholding of the principle of *secularism*. The state may be functioning with bureaucratic rationality, it may also be free of the domination of the clergy, but it may still not recognize its citizens' freedom of religion and conscience. Although these are not contradictory but complementary processes, the secularization of state administration does not guarantee the recognition of individual freedom of belief.

In pre-modern West, temporal political authority was dominated by the Church, from which it sought independence during the establishment of the modern nation-states. In this process, Protestantism played an important part in breaking the clerical authority of the Catholic Church. The rise of Protestantism did not mean the decline of religiosity (Gorski, 2000). On the contrary, while the Catholic clergy abused their power and prioritized serving their own worldly interests and of those around them, Protestants embraced a more puritan and fundamentalist conception of religion. As opposed to the dominance of the Catholic Church over territorial powers in pre-modern Western Europe, the state dominated religious authority in Eastern Christianity. The Islamic Ottoman Empire followed the same tradition, subordinating religious organization and the clergy to political authority. State and religion were unseparated in this structure as well, but political rather than religious authority was dominant.

Religious identity continues to be important as culture and tradition in the modern nation-states of Western Europe, but the separation between religious and political authorities has largely been realized and the citizens' freedom of religion and conscience has come under protection. However, in Turkey, and in the Balkan nations which observe Orthodox Christianity and were parts of the Ottoman geography, the intertwining of state and religion is much higher than in Western European nations. States have explicit or implicit religious identities and, even if they are secularized in terms of bureaucratic functioning, they delineate and circumscribe religious freedoms from above and have limited or no tolerance for different religious identities in society. Or, more accurately, such tolerance has tended to expand, thanks to various ECtHR decisions faced by these states in the course of their joining the Council of Europe and/or the European Union (EU). It might be noted that Turkey, which has yet been unable to join the EU and does not take the ECtHR decisions very seriously, is not a part of this trend.

These observations bring us to a frequently heard argument about secularism in Turkey. The Kemalist republican project of modernization is often accused of establishing a regime that suppressed religion and religiosity. Mostly articulated by Islamists, but also supported by some liberals, the argument is that the Turkish *laiklik* (after the French *laicité*) is a radical form of secularism, involving state “domination” over religion, whereas a more liberal secularism would necessitate a “separation” between them. This thesis contains a number of fallacies examined in the following pages. Most basically, if this thesis were valid, it would be necessary to pronounce the Ottoman Empire just as secular as modern Turkey, because the republican state essentially inherited with some modifications the Ottoman institutions of regulating religious affairs. We may now expand on this point.

## Contesting Secularism

In the 1980's and 1990's, Islamists in Turkey, supported by some liberals, used to timidly and indirectly object to the constitutional principle of secularism by framing their argument in terms of the need to replace Turkey's “radical” secularism with a more “liberal” variant. At that time, the Foucaultian critique of secularism was also gaining popularity around the world. According to Asad, a leading name in this field, the problem originated from modernity itself, because the modern secular state had demarcated religion from the top-down by drawing boundaries around it. The modern state exercised its sovereignty to shape religion by removing it from the public sphere and forcing it into the private, thereby violating its own claim to neutrality and distance (Asad, 1993, 2003).

Asad would have been justified had he simply said that the modern state violates its own assertion to be liberal when it engages in illiberal practices, as it occasionally (or frequently) does. But his critique of secularism goes beyond this observation to the point of actually rendering it impossible to analyze such states as Saudi Arabia or the Islamic Republic of Iran. Although these states have not removed religion from the political sphere to confine it to the private, they do exactly what Asad accuses the modern-liberal state of doing. They define correct religion, stipulate or set limits on religious practices, distinguish between proper and improper faiths, and discriminate against citizens of different faiths. All of this also took place in the pre-modern Ottoman Empire. If we were to adopt Asad's thesis, we would have to define these states as secular as well.

According to Asad, the intervention of the secular state in effect creates a previously non-existent category named “religion,” which can only be exercised in the private sphere. Therefore, what is liberated by this presumably “liberal” state is already in a clearly delineated form. The implication of this point seems to be that while religion covered the entire complexity of life before modernity, it has now been reduced to being defined and recognized only in its private aspect. The desire expressed by this critique is clear: the phenomenon that has been separated out as religion and forced into the private sphere should be brought back into the center of social and political life, so that it could re-encompass it entirely.

In fact, the religious institutions where secularism prevails, such as the Church in the Christian West, seem to have consented to their place in the political system. But the desire that is implicit in Asad's critique is openly expressed by Islamists, often in Western countries and by challenging the existing secular arrangements, such as in their demand that civil status laws conform to religious rules. But political and legal arrangements that aim to align people according to their religious belongings and to classify different faiths as legitimate or illegitimate have existed both before and after modernity; and states that pursue these aims are neither secular nor liberal. Therefore, the theses of Asad and his numerous followers lack coherence. They seem to be mostly motivated by the ideological concern that the modern-secular state has distanced religion, and its organized institutions, from political involvement.

However, there is a good reason for this exclusionary arrangement. While religious regimes consider certain beliefs illegitimate and persecute those who hold them, liberal/secular states (*normatively*) do not do so and allow everyone to have equal freedom of belief. Which is why they assign religion to the individual conscience of citizens and leave them free in the private sphere, but clear the commonly shared public sphere from the sway of belief systems. The involvement of organized belief systems in politics would create the danger of granting priority to the demands of the adherents of the majority (or otherwise dominant) faith at the expense of the others, potentially leading to mistreatment in cases of conflict. The preservation of a liberal order depends on a secular arrangement where religious beliefs are kept out of the public sphere. If in a purportedly liberal and secular regime there are practices of religious discrimination, or violations of religious freedoms, the source of the problem is not secularism *per se*, but rather an improper or inadequate implementation of it. This seems to be the case in France (Akan, 2017b), although this point deserves a separate treatment. Turkey on the other hand definitely fits this description, considering the degree of continuity between the Ottoman Empire and the Republic of Turkey with regard to the institutions that regulate state-religion relations.

The Republic of Turkey, defined as “secular” in its constitution and described as radically or rigidly so by Islamists and postmodern multiculturalists, actually is and has always been far from it. This can readily be seen by looking at two basic features of the state, present from its foundation: Islam is central in the definition of national identity, and the Islamic bureaucracy, called the Presidency of Religious Affairs (*Diyanet Işleri Başkanligi*, DIB), occupies an important place within the organization of the state. In the extant literature, the DIB is often portrayed as a tool for bringing religion under control and as such the main instrument of “oppressive” secularism. We have seen that this interpretation is conceptually flawed and it can easily be shown to be factually inaccurate as well. It would be more accurate to describe the DIB as a tool of state control over society through religion than a tool of state suppression of religion.

In order to see that religion is not excluded from politics in Turkey despite the constitution and other relevant legislation, we do not need to reflect on the currently ruling Justice and Development Party (*Adalet ve Kalkinma Partisi*, AKP) or its predecessor, the Welfare Party (*Refah*), as the first examples that come to mind. Even if we leave aside the fact that religious orders and brotherhoods, nominally banned since 1925, have always played a role in political mobilization, just remembering the daily use of such political slogans as “serving the people is serving God” or “the flag will never be lowered and the *ezan* [call to prayer] will never be silenced” makes one wonder how compatible they are with a secular regime. The complaint that religion in Turkey is under state domination, and therefore freedom of religion is hindered by the “secular” state, is often made by sections of the Muslim majority. However, while it is true that religious freedoms are restricted in Turkey, this applies not to the Muslim majority, but to other (officially recognized or unrecognized) faith communities that actually need the protection of the secular state.

## Nation and Religion

According to Article 66 of the Turkish constitution, “Everyone bound to the Turkish State through the bond of citizenship is a Turk.” But in popular culture, and even in some dealings with state institutions, non-Muslim citizens of Turkey are not recognized as Turks. Kurds, who complain of being excluded by the mentioned constitutional article, are considered Turkish because they are Muslim. Or, more precisely, their self-definition as Kurds is not tolerated, and in a sense Turkishness is imposed on them. By contrast, non-Muslims can never enter the realm of Turkishness and suffer from various forms of discrimination, even though nominally they are equal citizens. Anyone, from an ordinary person to a high-ranking official, may routinely make derogatory references to non-Muslims or ostracize them in social contexts. A systematic discrimination may also be observed in the formal functioning of the state, whereby citizenship rights of non-Muslims are curtailed in violation of relevant laws and international conventions (Ekmekcioglu, 2014). An evaluation of some examples below will reveal that the Turkish state, described in the pre-AKP period as “rigidly secular” by Islamists and some liberals, has not been secular (properly understood) either before or (obviously) after the AKP. While the dominant faith community is organized within the state, the freedom of citizens outside of this community is clearly circumscribed.

It may be considered essentially wrong for a secular state to have the authority to determine which faith community will or will not be officially recognized. But the practice exists in many countries that better qualify as secular than Turkey. This occurs for some concrete reasons, including the need to obtain zoning permits for the construction of houses of worship, have separate sections in cemeteries and days off on religious holidays, and so on. States with this practice determine and declare the criteria for officially recognizing a faith community, such as its size, the public safeness of its rituals, and the like. They then typically assign the recognized group a legal personality in order to regulate formal relations with it within the context of laws and to have an interlocutor in case of problems. Provided they are objective and reasonable, to specify such criteria for recognition and to expect the group to obey the relevant stipulations do not contradict the logic of secularism, because they significantly contribute to the facilitation of public services to these communities. The important point is that the state remains neutral among religions, does not dispute the legitimacy of the belief, and opens up space for individual freedoms without being intrusive.

However, the situation in Turkey is very different from this. There is no practice of recognizing or not recognizing a faith community according to any objective set of criteria. There are some officially recognized religions, but only by force of international treaties. Although officially recognized religious communities are granted certain rights, the actual practice of recognition is narrower than envisaged by the treaties in question. Besides, these communities do not have legal personality. There are also religious groups that are not officially recognized but their existence is tolerated. Worse still, the state distinguishes between legitimate and illegitimate religions. Therefore, alongside the ordinary culture of discrimination among the people, the state arbitrarily favors certain religions or sects and is systematically biased against others, all of which is in violation of national laws and international treaties.

Ask anyone with a basic “civic education” in Turkey about which religious minority groups are protected by the Lausanne Treaty (1923), the document that confirmed Turkey's independence, and they will recite the following three: Armenian, Jewish, and Greek Orthodox. But these groups are not mentioned by name anywhere in the “Protection of Minorities” section of the Treaty (Articles 37–45). The rights specified in this section belong to every non-Muslim citizen; yet the state has arbitrarily limited the non-Muslim category to these three groups, possibly following the traditional *millet* system of the Ottoman Empire. Even then, it has not fully endowed them with the stated rights (Oran, 2007). For example, Article 39 clearly states: “Differences of religion, creed or confession shall not prejudice any Turkish national in matters relating to the enjoyment of civil or political rights, as, for instance, admission to public employments, functions and honors, or the exercise of professions and industries.” Yet no non-Muslim citizen can be found in any high-ranking official capacity in the government bureaucracy or the armed forces, even if they belong to one of the formally recognized groups.

Or, take the case of Syriacs. Article 40 states: “Turkish nationals belonging to non-Moslem minorities … shall have an equal right to establish, manage and control at their own expense, any charitable, religious and social institutions, any schools and other establishments for instruction and education, with the right to use their own language and to exercise their own religion freely therein.” But, although Syriac churches actually exist, it has been impossible for the Syriac community (unlike the Armenian, for example) to open their own schools. Those community schools that can be opened, on the other hand, are subject to the same legislation as any “private” school, with curtailed rights compared to public schools. The dominant religious group, by contrast, is organized within the state structure. The DIB's budget comes from taxes paid by all citizens, belonging to any or no belief system, and covers the expenses of mosques and salaries of the clergy. All other religious communities have to cover their expenses from their own resources.

The lack of legal personality is outside of the European norm, and creates financial and administrative problems for even the officially recognized religious groups (Bottoni, 2013). The current legal framework does not allow the Armenian or Greek Patriarchates or the Jewish Chief Rabbinate to acquire property, employ staff, or be a party in court. The affairs of the congregations are managed through associations and foundations, subject to legislation applicable to any such organization. The communities lack the opportunity to coordinate their internal affairs according to their own religious principles and are exposed to arbitrary government interventions. The state has been able in the past to confiscate the properties of “minority” foundations simply on the basis of governmental decrees or judicial decisions, and they were only returned through ECtHR judgments years later. Likewise, the state has been, and still is, able to arbitrarily intervene in the elections of community leaders by simply issuing regulations or directives. At the same time, some religious groups are permitted to establish themselves in an indirect but precarious manner in the form of associations, without the need for official recognition. Numerous Protestant churches are in this category of being allowed to exist without any assurances.

## Minority Religions

In addition to the officially recognized and unrecognized religious communities mentioned above, there are also those communities of faith that the state knows of and tolerates, but does not consider their faiths as “legitimate” religions. The best known among them is the Alevi community, discussed briefly below. But first, for a different example, we may address the status of the Baha'i community. With ~5 million adherents worldwide and recognized in most countries, the Baha'i faith is still seen as a problem in some. Particularly in the Islamic Republic of Iran, the community has been subject to persecution. The Baha'is in Turkey are currently in the “tolerated” category. But in many instances from the early republican years until recently, they have been raided during gatherings in homes and arrested for sentencing, as if they were engaged in an illegal activity. More interestingly from our point of view, Turkish courts have debated the authenticity of the Baha'i faith, as if this were a matter that concerned a secular state. In a court case in the early 1960's, while the Baha'is argued that their belief system was a distinct and “independent” religion, expert opinion received from the DIB rejected this claim and portrayed the Baha'i faith as a (deviant) sect or order (Özşuca, 1997).

An explicit presentation of the “official” view may be found in the book on the Baha'i faith by Figlali (1994), a professor who served as dean of theology school at a public university in the 1980's. The book was published by *Türkiye Diyanet Foundation*, described on its website (https://tdv.org/en-EN/) as having been “established to support the activities” of DIB. Figlali may be considered the foremost authority on the subject as far as DIB is concerned, for he is also the author of the article on Baha'ism in the same foundation's *Encyclopedia of Islam* (https://islamansiklopedisi.org.tr/bahailik).

According to Figlali, “it is possible to see all the traces of ancient Iranian religions and civilizations” in the *esoteric* mentality, which has been the most “divisive current” for Islam (Figlali, 1994, p. IX, *my translation*). He adds: “Babism and Bahaism are also a current of mischief that emerged in the nineteenth century from mysticism tangled up with esoteric interpretations and Mahdism, which is one of the main principles of Shi'ism” (p. X). According to this author, “Bahaism can be called a perverted order within Shi'ism, or it can also be said to be one of the perverted sects among those that originated from Islamic culture but moved outside of the circle of Islam” (p. 91). In the author's assessment, “The Turkish Court of Cassation rightly confirmed in an extraordinarily fair and scientific decision [in 1962, see above] … that Bahaism cannot be considered a separate religion and cannot be accepted as such. Thus, they [Baha'is] could not attain minority rights and are therefore under prosecution in accordance with Turkish laws, as a perverted sect and a secret religious society” (p. 92). According to Figlali, that is how it should be, because “Bahaism constitutes the last stage of historical intrigues against Islam; because, as you can see, it started with the destructive esoteric movement, served and still serves as a tool of the Zionist and Crusader world and the imperialists” (p. 92).

One might be tempted to say that if there is freedom of thought and belief, anyone should be able to put forward any thoughts on any belief system. But there is a striking inequality in the “freedom of expression” here. Turkish Penal Code (Article 216) explicitly prohibits the incitement of hatred or hostility against, and the public denigration of, “a section of the public on grounds of social class, race, religion, sect, gender or regional differences.” Moreover, a new section added to this article in 2004 (during AKP rule), bans the public denigration of “the religious values of a section of the public,” which has caused the conviction of numerous people by allegations of crimes against Islam. Even if there were no such prohibitions, it is clear that such statements about minority faiths as those quoted above, especially when uttered by people in official positions of authority, are oppressive and intimidating.

The Baha'i community in Turkey, with only a few thousand members, may not be perceived as a serious cultural or political threat. But another religious minority that seems to embody Figlali's concept of a “perverted sect” and a “divisive movement” of Iranian origin is the Alevi community, estimated (because they are not counted in the censuses) to constitute nearly 20 percent of the population. Alevis' relationship with Islam is often seen as dubious or ambivalent, much like the Kurds' relationship with “Turkishness.” The dominant assumption is that Alevis do not understand Islam correctly or do not follow its tenets properly. We have repeatedly noted that the specific doctrines of a religious community should not concern a secular state, provided they do not pose a threat to public order or to human rights and freedoms. But the ongoing discrimination against Alevis actually originates from political causes and is inherited from the Ottoman era. Indeed, from the Ottoman times to the present, the primary factor that determines state-religion relations in Turkey has been the state's political priorities. The state has been prone to dominate or manipulate religious identities, as well as their relations with the state and with each other, for the purpose of power.

## Ottoman Legacy

Belying the honorific title of “*ghazi*” (holy warrior), the founding dynasty of the Ottoman Empire actually had heterodox Islamic beliefs and easily entered into political alliances with non-Muslim neighbors for the purpose of conquest. This situation was more manifest when the dynasty was a frontier principality. After the conquest of Istanbul, however, the process of building state institutions led the political elites to adopt a more literate Islam. Although heterodox and *Sufi* beliefs continued to abound among the people (and members of the dynasty), the official identity and ideology of the state veered toward Sunni orthodoxy.

Sultan Mehmed II (1451–1481), who conquered Constantinople in 1453 with the support of *pashas* of Christian origin, initially claimed the title of “Caesar of the Roman Empire.” The Ottoman state, which ruled mostly in the Thrace before capturing Istanbul, already had a large non-Muslim population. After the conquest, the foundations of the Ottoman “*millet*” system were laid, whereby the state collected a higher rate of tax from the “people of the book” (i.e., Christians and Jews), but did not interfere in their religious freedom and allowed them some autonomy to run their communal affairs.

The power of the Muslim *ulema* rose among state elites during Mehmed II's rule. But most critical for the establishment of orthodox Sunni identity was the rivalry and conflict with the Safavid state of Iran, during the reign of (Yavuz) Sultan Selim (1512–1520). After settling in Istanbul and aiming to expand eastward into farther reaches of Anatolia, the Ottoman state faced the Safavids, a Shiite dynasty of Turkish origin which also tried to expand into Anatolia. In the battle of *Chaldiran* in 1514, Sultan Selim defeated Shah Ismail, the founder of the Safavid state, and put an end to his ambitions to expand westward. The rebellions of the Turcoman tribes that were known as the “*Kizilbaş*” (because of their distinctive red headgear), and accused of supporting Shah Ismail, were violently suppressed.

After defeating Shah Ismail, Selim had the opportunity to advance toward the south, conquered Egypt in 1517 and thus ended the Mamluk state, which held the post of Caliph. It is historically unclear whether Sultan Selim actually took over the seat of Caliphate and transferred it to the Ottoman dynasty and, if he did, whether this seat had any real significance for the Ottomans until the nineteenth century. At any rate, what is clear and important from our point of view is that Sunni identity won absolute hegemony within the state in this period (Barkey, 2008). From then on, the “tolerance” shown to non-Muslim communities (provided they were “people of the book”), was denied to groups leaning toward heterodox Islam. Religious practices incompatible with Sunni orthodoxy came under discrimination and oppression. The community that today is collectively known as “Alevi,” despite an internal lack of homogeneity in terms of proximity to Islam and Sunni orthodoxy, descends from the *Kizilbaş*, and the discrimination they continue to experience originates from this period. The Ottoman state did not try to convert non-Muslims, except those individually recruited to serve in the army and the bureaucracy (the so-called “*devşirme*”), but made a special effort to convert non-Sunnis by using both pressure and incentive methods.

There is no question that this trend continued in the Republican period. The tentative move from an Islamic to a national identity provided the Alevis with some relief at first. There was even an effort to discover within Alevi culture some elements that would underpin nationalist themes such as “real” Turkish-Islam or the legacy of Central Asian traditions (Dressler, 2013). However, having concealed their identity for safety among the Sunni majority and having been subjected to occasional mass violence (particularly in the 1970's, in the guise of political conflict between left and right), the Alevi community could only begin to demand public recognition in the 1990's, within the context of the rise of identity politics (Göner, 2005).

## Is Turkey Secular?

Historian Ocak (1998, p. 95–96) observes that religion and the clergy were at the service of state power in the Ottoman Empire: “In Ottoman official ideology, Islam materializes as a political tool of the central government. … Islam in the Ottoman State … is not outside the state and in a dominant position over the state, but under state control and dependent on the state” (*my translation*). This being the case, “the main task of the ulema … [as] a part of the state … was to legitimize all the acts and actions of the political power mechanism, in which the sultan occupied the center. Thus, the ulema were literally turned into a kind of religious bureaucrat” (p. 94). These descriptions exactly parallel the statements of the critics of the Republican regime, who assert that the state controls and suppresses religion and the religious people through the DIB (for details, see Gülalp, 2017). The portrayal of the Republic in this literature is not different from the portrayal of the Ottoman Empire by Ocak (or by Inalcik, 1989). Conceptually, this leaves us with a choice: either the Ottoman Empire was secular like the Turkish Republic or (more realistically) the Turkish Republic is not actually secular.

In Turkey's current constitution, the provisions on secularism are full of contradictions. For example, Article 24 envisages freedom of religious belief, but also mandates religious and moral instruction in schools. The same article rules that no one can be compelled to disclose their religious beliefs, but the state records the religious identity of every citizen routinely as Muslim, unless they are able to demonstrate their belonging to an officially recognized religious minority. Article 136 paradoxically states that the Presidency of Religious Affairs (DIB) shall exercise its duties “in accordance with the principles of secularism.” The impossibility of this contradictory task is evident in the code that describes the DIB's formal duties. The first article of Law No.633 indicates that the DIB was established “to administer the affairs concerning faith, worship and moral principles of the Religion of Islam, to enlighten society about religion, and to manage the places of worship.” No wonder, under AKP rule, the DIB has significantly enhanced its function of contributing to the hegemony of Islamist thought (Akan, 2017a, p. 239–243).

The AKP came to power having won the support of some liberal and democratic circles, in addition to Islamists, with a promise to expand religious freedoms and soften the allegedly “rigid” and “oppressive” secularism of the Kemalist state. Rejecting the anti-European stance of its predecessor, the Welfare Party, it started membership negotiations with the EU in its first years in office. Previously confiscated properties of non-Muslim foundations were returned thanks to ECtHR judgments that came down in this period. All this gave the impression that the AKP government defended freedom of religion not only for the Muslim majority but also for non-Muslims. The following years showed that this impression did not quite reflect the reality. The most recent and striking event has been the conversion of Hagia Sophia back into a mosque. Built in the 530's (AD) as the state cathedral of the Roman Empire, Hagia Sophia was made a mosque upon the conquest of Constantinople. The Kemalist government turned it into a museum in 1935, which was how it stayed until the summer of 2020. Then, in the course of the pandemic, and disregarding the risks involved in gathering crowds from all parts of Turkey for the first prayer and celebration, the AKP government declared the edifice to be a mosque again. Other problems inherited by the AKP and left unresolved included the status of the Halki Seminary (the Greek Orthodox Theological School in Istanbul), which was closed in 1971 and never reopened despite endless negotiations and promises, and the recurrent practice of intervening in the election of the Armenian Patriarch. But the real test for the AKP government was its attitude toward Alevis. To a set of initiatives called “democratic openings,” the AKP government added an “Alevi opening,” which consisted of a series of workshops during 2009–2010 presumably to identify Alevi grievances and demands. The “opening” started with a bang, but ended with a whimper. The government minister responsible for the event simply announced at a press conference in March 2011 that Alevi demands would largely go unmet.

Much has been written on this episode, so two brief remarks will suffice. First, it was not necessary to occupy the public opinion with such an effort (show?) for months on end, because Alevi demands were already very clear and easy to meet. Despite the theological and/or political differences within the Alevi community (a normal occurrence overstated by AKP circles), they all agree on the simple need to have the Alevi faith accepted as a “legitimate” belief system. Secondly, and more interestingly, the AKP government based its rejection of Alevi demands on the institutions often regarded as elements of the “Kemalist state's oppressive secularism.” The rejection of the Alevi *cemevi* as a house of worship, for example, was based on the 1925 prohibition of religious orders and brotherhoods (a legal code that is still in the books but rarely implemented) and the opinion of theologians at the universities and in the DIB.

## Concluding Thoughts

Turkey has been rapidly declining in democracy and human rights indexes in recent years, but the AKP government, continuously in power since the end of 2002, never tires of declaring that democratic rights and freedoms have significantly improved under their rule. Is this simply a case of trying to deceive people via “*post-truth*” methods, in the fashionable phrase that mystifies lying? Is there no point of contact between propaganda and reality? Even if there were not any, why would a government that has restricted freedoms in multiple ways resort to such propaganda?

Some clues may be found in the frequent resurfacing of the outdated “headscarf” debate, which was reignited on several occasions just during the 2 months in which this article was being prepared. In December 2020, innumerable essays appeared on the *Netflix* series “*Ethos”* (aired in Turkey as “*Bir Başkadir*”), about a young, conservative, working class woman, who wears a headscarf and experiences urban prejudice. The show attracted far more attention than any other TV series, despite its numerous flaws originating from ignoring the current social and political realities of Turkey. In the same month, Fikri Saglar, a former deputy of the Republican People's Party, stated on a TV panel that he would doubt the impartiality of a judge wearing an Islamist headscarf, if he were to stand trial before her. This statement caused an uproar. AKP spokespeople accused him of fascism, racism, barbarism, and so on, and the public prosecutor of Ankara started an investigation. In January 2021, Ali Babacan, a former cabinet member of the AKP government, currently leading a minor opposition party, burst into tears during his address to the party congress when he mentioned that her sister could not attend university because of her headscarf. Also in January, the controversial appointment to the elite Bogaziçi University of an underqualified AKP supporter as the new rector led to a public discussion about how this university condoned headscarves in the past, as if that were the sole or primary criterion of a university's worth.

AKP leaders did not make inflammatory remarks like the Welfare Party leader Necmettin Erbakan, who vowed back in 1996 as prime minister that university rectors would be made to stand salute to the girls wearing headscarves. But they still placed the headscarf issue at the center of their ideological campaigns, at first discreetly, but later more explicitly. It may be surprising that the headscarf issue became so central, considering the wide variety of human rights violations in Turkey. The configuration that made it a suitable political thesis for that period emerged out of a combination of several factors. After wavering for a while at the end of the Cold War, NATO and the Western Bloc decided to focus on the threat posed by the anti-Western Islamist movements that had gathered pace since the 1980's. Fundamentalism replaced communism as the new global enemy. In this context, Turkey, as a NATO member and (on paper) a “secular and democratic” country notwithstanding its Muslim-majority population, was charged with much responsibility. Geographically positioned at the frontline against the Eastern Bloc during the Cold War, Turkey was located at the forefront of this new confrontation as well. Turkey's thesis for joining the EU in the 1990's was the looming threat of fundamentalism inside the country. If left out, it was argued, Turkey might be unable to prevent the fundamentalists from taking power, and therefore Islamist political symbols displayed in the guise of freedoms had to be fought. The headscarf was the most visible of these symbols. Widely used at that time in schools and universities, considered to be the natural bastions of science and secular thinking, the headscarf was seen as a concrete indicator of the danger of a “clash of civilizations,” both in Turkey and in parts of Europe with large Muslim populations.

Much litigation took place against the headscarf ban in various national courts and the ECtHR. But the issue was often presented as a *political* matter of “religion vs. secularism” rather than one of individual freedom, leading the judges to perceive it as such, which rendered the problem largely unsolvable (Gülalp, 2019). The clothing issue in Europe, symbolized by the headscarf but including the veil, burkini, etc., is still unresolved and continues to bolster the Islamist theses on Islamophobia and victimization. In Turkey, however, the AKP government in a sense resolved the headscarf problem, but only at the expense of many new problems. The AKP was initially self-described as “conservative democratic” and therefore seen as a concrete case of the “moderate Islam” option promoted especially after 9/11. AKP's (unfulfilled) promise of “freedom of religion” was falsely equated with the development of democracy. But freedom of thought, belief and conscience does not mean opening up space for religions; it simply means the freedom of individuals to have any ideas and beliefs as they wish. Making space for religion in politics leads to the restriction of freedom of thought and conscience.

In fact, Islamic identity as a means of solidarity has been more important for the AKP government than the rules and principles of Islam. Marking out a lifestyle community in order to create a “common people vs. the elite” dichotomy has been an effective way of building political power. No doubt, such a political project can be based on any identity. Moreover, identity politics may have a progressive function as an opposition movement by helping to uncover the structural conditions that create social inequalities between groups. But they are certainly not a democratic basis on which a project for power could be built. An identity movement that achieves power, or even on the way to power, is bound to be exclusionary and undemocratic. It is by definition intolerant of diversity and individual freedoms. Among the possible types of identity politics, perhaps religious identity is the most distant to democracy, because religious creed is considered sacred. Even though it is flexible in reality and open to interpretation depending on time and place, it will still be deemed absolute and unquestionable when put forward by an authority. The most suitable political ideology to support an authoritarian regime seems to be the one based on religious identity.

## Author Contributions

The author confirms being the sole contributor of this work and has approved it for publication.

## Funding

The article was prepared in the context of the GREASE project, funded by the European Union's Horizon 2020 research and innovation programme under grant agreement number 770640.

## Conflict of Interest

The author declares that the research was conducted in the absence of any commercial or financial relationships that could be construed as a potential conflict of interest.

## Publisher's Note

All claims expressed in this article are solely those of the authors and do not necessarily represent those of their affiliated organizations, or those of the publisher, the editors and the reviewers. Any product that may be evaluated in this article, or claim that may be made by its manufacturer, is not guaranteed or endorsed by the publisher.
